# Enhanced 1,3-propanediol production with high yield from glycerol through a novel *Klebsiella*–*Shewanella* co-culture

**DOI:** 10.1186/s13068-023-02304-4

**Published:** 2023-03-24

**Authors:** Yanxia Wang, Zijian Wan, Yueting Zhu, Haibo Hu, Yujia Jiang, Wankui Jiang, Wenming Zhang, Fengxue Xin

**Affiliations:** 1 College of Food Science and Light Industry, Nanjing Tech University, Nanjing, 211800 People’s Republic of China; 2 State Key Laboratory of Materials-Oriented Chemical Engineering, College of Biotechnology and Pharmaceutical Engineering, Nanjing Tech University, Puzhu South Road 30#, Nanjing, 211800 People’s Republic of China; 3 Jiangsu National Synergetic Innovation Center for Advanced Materials, Nanjing Tech University, Nanjing, 211800 People’s Republic of China

**Keywords:** *Klebsiella*, 1,3-Propanediol, Glycerol, Cofactor regulation, Microbial co-cultivation system

## Abstract

**Background:**

1,3-Propanediol (1,3-PDO) is a platform compound, which has been widely used in food, pharmaceutical and cosmetic industries. Compared with chemical methods, the biological synthesis of 1,3-PDO has shown promising applications owing to its mild conditions and environmental friendliness. However, the biological synthesis of 1,3-PDO still has the problem of low titer and yield due to the shortage of reducing powers.

**Results:**

In this study, *Klebsiella* sp. strain YT7 was successfully isolated, which can synthesize 11.30 g/L of 1,3-PDO from glycerol in flasks. The intracellular redox regulation strategy based on the addition of electron mediators can increase the 1,3-PDO titer to 28.01 g/L. Furthermore, a co-culturing system consisting of strain YT7 and *Shewanella oneidensis* MR-1 was established, which can eliminate the supplementation of exogenous electron mediators and reduce the by-products accumulation. The 1,3-PDO yield reached 0.44 g/g and the final titer reached 62.90 g/L. The increased titer and yield were attributed to the increased redox levels and the consumption of by-products.

**Conclusions:**

A two-bacterium co-culture system with *Klebsiella* sp. strain YT7 and *S. oneidensis* strain MR-1 was established, which realized the substitution of exogenous electron mediators and the reduction of by-product accumulation. Results provided theoretical basis for the high titer of 1,3-PDO production with low by-product concentration.

## Introduction

1,3-Propanediol (1,3-PDO) is a versatile platform chemical, which can be used to synthesize polyester, polyurethane and heterocyclic compounds. Additionally, 1,3-PDO is also widely used in the fields of food, medicine, cosmetics and biodegradable plastics [1–3]. For example, 1,3-PDO can be used as a monomer to synthesize biodegradable polyester plastic—polytrimethylene terephthalate (PTT) [4, 5]. Currently, the chemical synthesis of 1,3-PDO was mainly originated from ethylene oxidation or propylene hydrogenation, which requires expensive catalysts and produces toxic intermediate compounds [6, 7]. In contrast, the microbial fermentation method has shown the advantages of renewability, green process and environmentally friendly property. To date, several microorganisms have been reported to produce 1,3-PDO including *Klebsiella*, *Clostridium*, *Citrobacter*, *Enterobacter* and *Lactobacillus* [8–16]. Among them, *Klebsiella* sp. has attracted great attention owing to its easy cultivation and mature genetic modification tools, indicating the promising potential for large-scale scaling 1,3-PDO production [1, 3]. Especially, 1,3-PDO-producing microbes show wild substrate spectrum, such as glycerol, glucose and other raw materials [17, 18].

As a reduced biochemical, the biological production of 1,3-PDO has the problems of insufficient supply of reducing powers [19]. In terms of the glycerol reduction metabolism, two NADH molecules are required for each molecule of 1,3-PDO synthesized from glycerol [20, 21]. Therefore, the classical anaerobic production of 1,3-PDO from glycerol is limited by the content of NADH. In *Klebsiella* sp., NADH required for the synthesis of 1,3-PDO through glycerol reduction metabolism is mainly regenerated during the oxidation of glycerol to pyruvate. Pyruvate can be further transformed into 2,3-butanediol (2,3-BDO), lactate, ethanol, acetate and other by-products [20, 21]. This process would competitively consume glycerol and NADH, thus affecting the titer and yield of 1,3-PDO [22, 23]. The other issue is the by-products formation, which will not only lead to the carbon loss, but also cause the toxicity towards microbial cells and inhibit the microbial growth [20, 21]. Therefore, to further increase the titer and yield of 1,3-PDO, it is necessary to maximize the utilization of carbon and reducing power.

Recently, synthetic microbial consortia have been widely used for bulk and fine chemicals production from renewable resources, which can perform more complicated tasks through the mutual interaction [24]. As known, electron transfer within different microbial species is a way to couple metabolic energy and co-factors balances, which also offers an efficient and cost-effective platform to directly control redox balances in bio-manufacturing system. However, few studies have addressed on the improvement of 1,3-PDO production by the microbial consortia instead of the supplementation of exogenous electron carriers [25].

In this study, a 1,3-PDO producing *Klebsiella* sp. strain YT7 was successfully isolated, which can synthesize 1,3-PDO from glycerol. Fermentation conditions will be further optimized to improve its 1,3-PDO production capability. To eliminate the supplementation of electron carriers, an artificial co-culturing system consisting of *Klebsiella* sp. strain YT7 and *Shewanella oneidensis* MR-1 was established.

## Results

### Isolation and phylogenetic analysis of *Klebsiella* sp. strain YT7

**Fig. 1:**
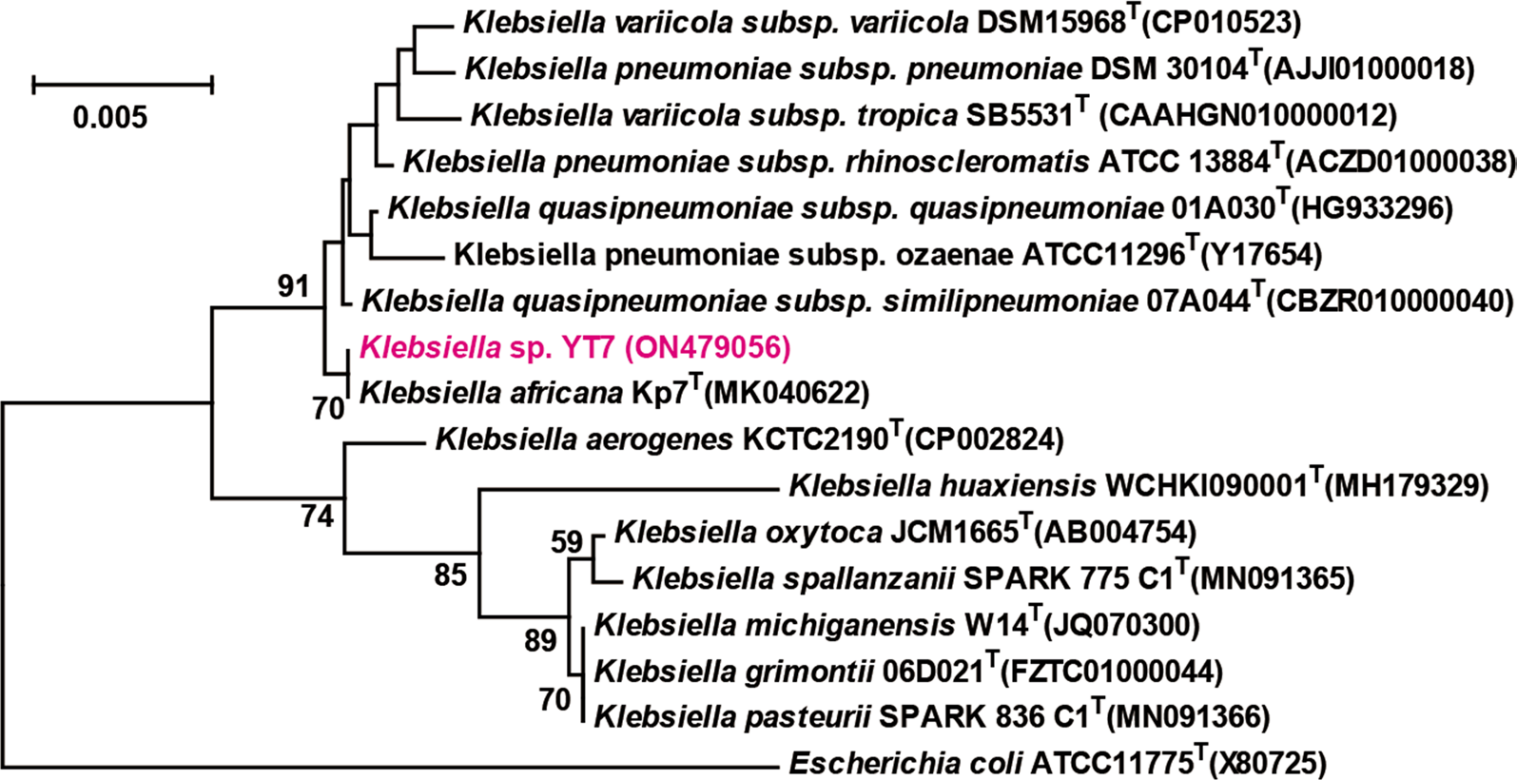
Neighbor joining tree based on 16S rRNA gene sequences, showing the relationship between strain YT7 and related taxa. The sequence of *Escherichia coli* ATCC11775^T^(X80725) was used as the out group. Bar, 0.005 substitutions per nucleotide position

Five strains with 1,3-PDO production capacity were screened from 20 isolated single colonies. After more than 5 consecutive cultivation, a strain named as YT7 was isolated, which can directly produce 11.30 g/L of 1,3-PDO from 40 g/L of glycerol. Moreover, 3.92 g/L, 5.41 g/L and 3.32 g/L of 2,3-BDO, ethanol and lactate were detected, respectively. The 16S rRNA gene sequence of strain YT7 (GeneBank NO. ON479056) is highly similar to *K. africana* Kp7^T^ (MK040622) (98.7%). The neighbor-joining phylogenetic tree reconstructed based on 16S rRNA gene sequences suggested that strain YT7 belonged to the genus *Klebsiella* (Fig. 1). Thus, strain YT7 was designated as *Klebsiella* sp.

### Optimization of fermentation conditions to enhance 1,3-PDO production from glycerol

**Fig. 2:**
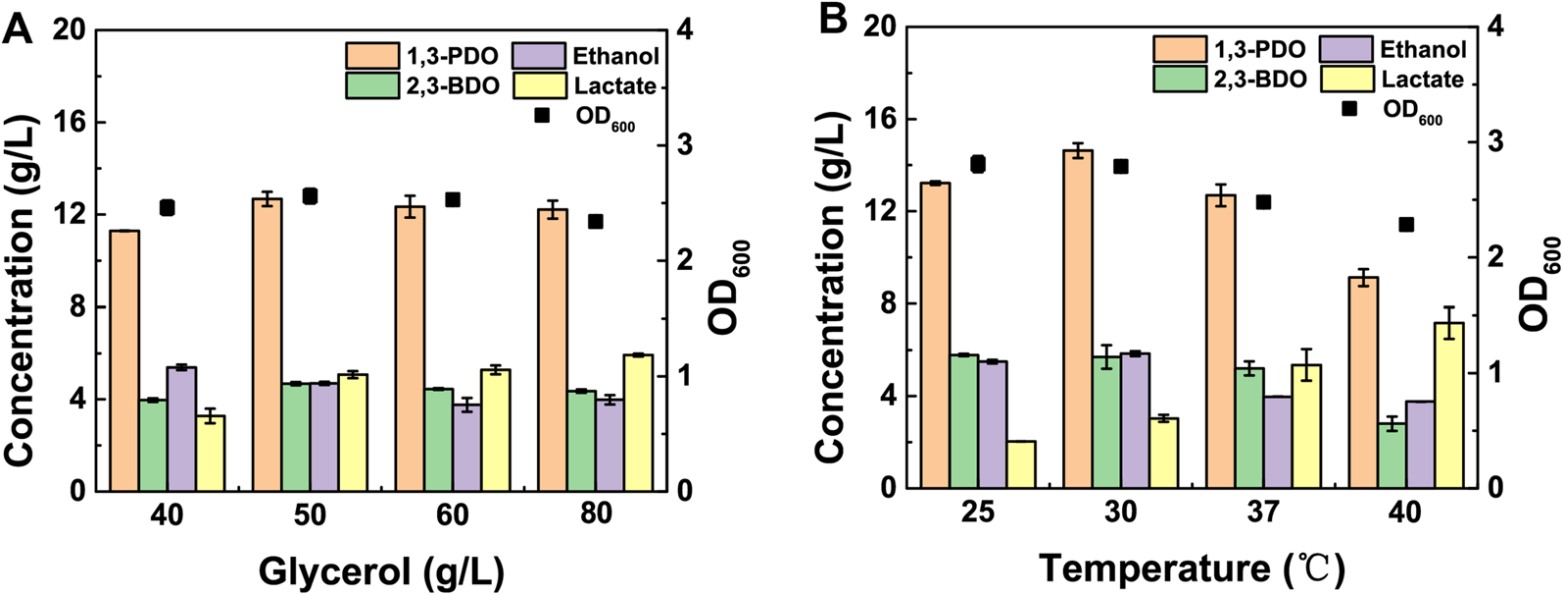
Optimization of fermentation conditions for enhanced 1,3-PDO production from glycerol for *Klebsiella* sp. YT7. **A** Effects of different initial glycerol concentration on 1,3-PDO production for strain YT7 in 37℃. **B** Effects of different fermentation temperature on 1,3-PDO production for strain YT7 from 50 g/L of glycerol

In order to further increase the 1,3-PDO titer by strain YT7, a series of fermentation conditions were accordingly optimized including initial glycerol concentration and fermentation temperature. When the initial glycerol concentration was set at 40 g/L, the 1,3-PDO titer was the lowest (11.29 g/L), while the by-product of ethanol was the highest (5.39 g/L) (Fig. 2a). When the initial glycerol concentration was increased to 50 g/L, the 1,3-PDO titer reached 12.68 g/L. When the initial glycerol concentration was increased from 50 to 70 g/L, the production of 1,3-PDO (12.68 g/L vs. 12.21 g/L), 2,3-BDO (4.68 g/L vs. 4.36 g/L) and ethanol (4.69 g/L vs. 3.98 g/L) were all decreased. On the contrary, the lactate accumulation was increased with the increase of initial glycerol concentration (Fig. 2a). Therefore, 50 g/L of glycerol was chosen as the optimal initial concentration and implemented in the subsequent experiments.

Four different fermentation temperatures including 25 °C, 30 °C, 35 °C and 40 °C were further investigated to detect their effects on 1,3-PDO production (Fig. 2b). Generally, strain YT7 preferred 30 °C for microbial growth. When the cultivation temperature was higher than 30 °C, the biomass of strain YT7 was significantly decreased. Meanwhile, strain YT7 generated the highest 14.63 g/L of 1,3-PDO at 30 °C from 50 g/L of glycerol. It should be noticed that the by-product of ethanol was increased with the increase of cultivation temperature, while 2,3-BDO and ethanol were decreased with the increase of cultivation temperature. For instance, when the temperature was maintained at 40 °C, the production of 2,3-BDO and ethanol was decreased to 2.80 g/L and 3.76 g/L.

Currently, the reported optimum growth temperature of *Klebsiella* was 30–37 °C. And most fermentation temperature for 1,3-PDO production by *Klebsiella* was set at 37 °C [26–28]. In this study, strain YT7 showed the highest 1,3-PDO production and relatively low by-products production at 30 °C. As known, glycerol dehydratase and 1,3-PDO oxidoreductase were key catalytic enzymes in the biosynthesis of 1,3-PDO. It was reported that the optimal temperatures for the enzymatic reaction of glycerol dehydration and 1,3-PDO oxidoreductase were 28–45 °C and 30–45 °C, respectively [29, 30]. Therefore, it is speculated that *Klebsiella* has a high ability to synthesize 1,3-PDO at 28–37 °C, and strain YT7 prefers 30 °C to synthesize 1,3-PDO.

### Regulation of redox co-factors based on electron mediators

The oxidation–reduction cofactor plays an important role in the metabolic activity of microorganisms, as it is not only an electronic receptor catalyzing the metabolic metabolism in cells, but also provides the restoration for certain oxidation reactions [31]. In terms of the reducing metabolic pathway, 1,3-PDO oxidoreductase can generate one molecular 1,3-PDO with the consumption of two molecules NADH [20, 21]. Therefore, the oxidation–reduction cofactor of NADH/NAD^+^ was further regulated by electron mediators to explore the effects of 1,3-PDO synthesis by strain YT7.

**Fig. 3:**
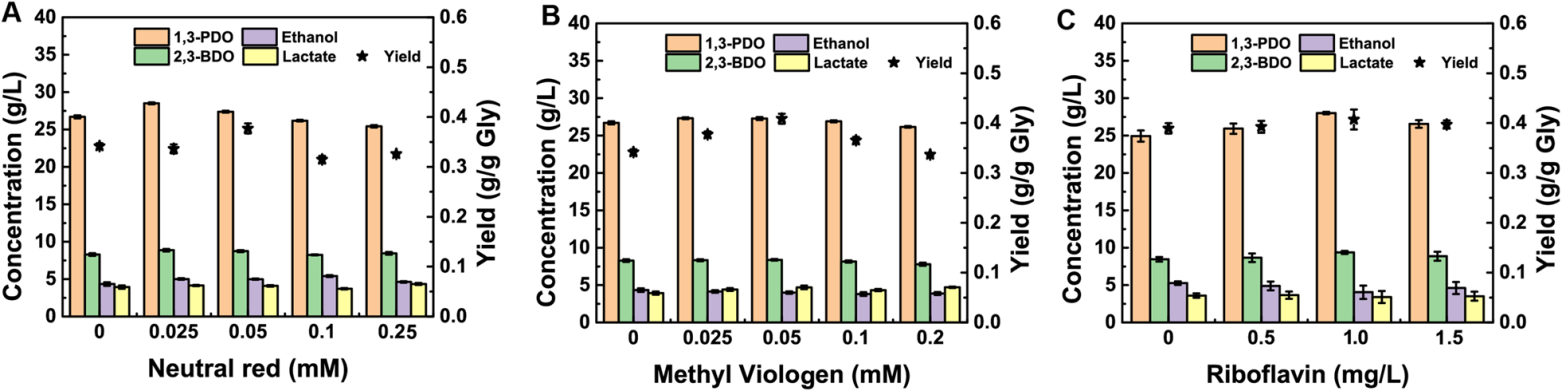
Effects of different electron mediator on 1,3-PDO production and growth for *Klebsiella* sp. YT7. **A** Effects of different neutral red concentration on 1,3-PDO production for strain YT7. **B** Effects of different methylviologen concentration on 1,3-PDO production for strain YT7. **C** Effects of different riboflavin concentration on 1,3-PDO production for strain YT7

The electron mediator can realize the electron transmission by carrying electrons to cross the cell membrane, which will affect NADH levels in microorganisms [32–34]. Accordingly, non-physiological (neutral red and methylviologen) electron mediator and physiological electron (riboflavin) mediator were used to explore their effects on 1,3-PDO biosynthesis by strain YT7. As seen in Fig. 3a, low concentration of neutral red increased the 1,3-PDO production. For example, when the concentration of neutral red in the medium was set at 0.024 mM, the highest 28.50 g/L of 1,3-PDO occurred, which was increased by 6.75% compared with the control group (Fig. 3a). With the increase of neutral red in the medium, the 1,3-PDO yield was decreased gradually (Fig. 3b). Compared with neutral red, the addition of methylviologen did not significantly improve the 1,3-PDO production, however, its yield was significantly improved (Fig. 3b). When the methylviologen concentration in the medium was set at 0.05 mM, the highest 0.41 g/g of 1,3-PDO yield from glycerol was achieved by strain YT7, which was 20.59% higher than the control group (Fig. 3b).

The addition of electron mediator riboflavin can also improve the titer and yield of 1,3-PDO synthesized by strain YT7. When the riboflavin concentration was set at 1 mg/L, 28.04 g/L of 1,3-PDO (yield was 0.41 g/g) was synthesized by strain YT7, which was increased by 12.48%. As seen in Fig. 3c, the production change of by-product 2,3-BDO was positively correlated with 1,3-PDO. Meanwhile, the by-products production of ethanol and lactate were the lowest (4.04 g/L and 3.40 g/L, respectively) with 1 mg/L of riboflavin. In strain YT7, the addition of riboflavin increased the carbon flux towards 1,3-PDO and 2, 3-BDO production and reduced the carbon flux towards ethanol and lactate production, which improved the 1,3-PDO yield (Fig. 3c). As known, neutral red and methylviologen belong to non-physiological electron mediators, which have toxicity to cells. Therefore, riboflavin was used in the fermentation production of 1,3-PDO by strain YT7 for further research.

### Construction of two-bacterial co-culturing system

**Fig. 4:**
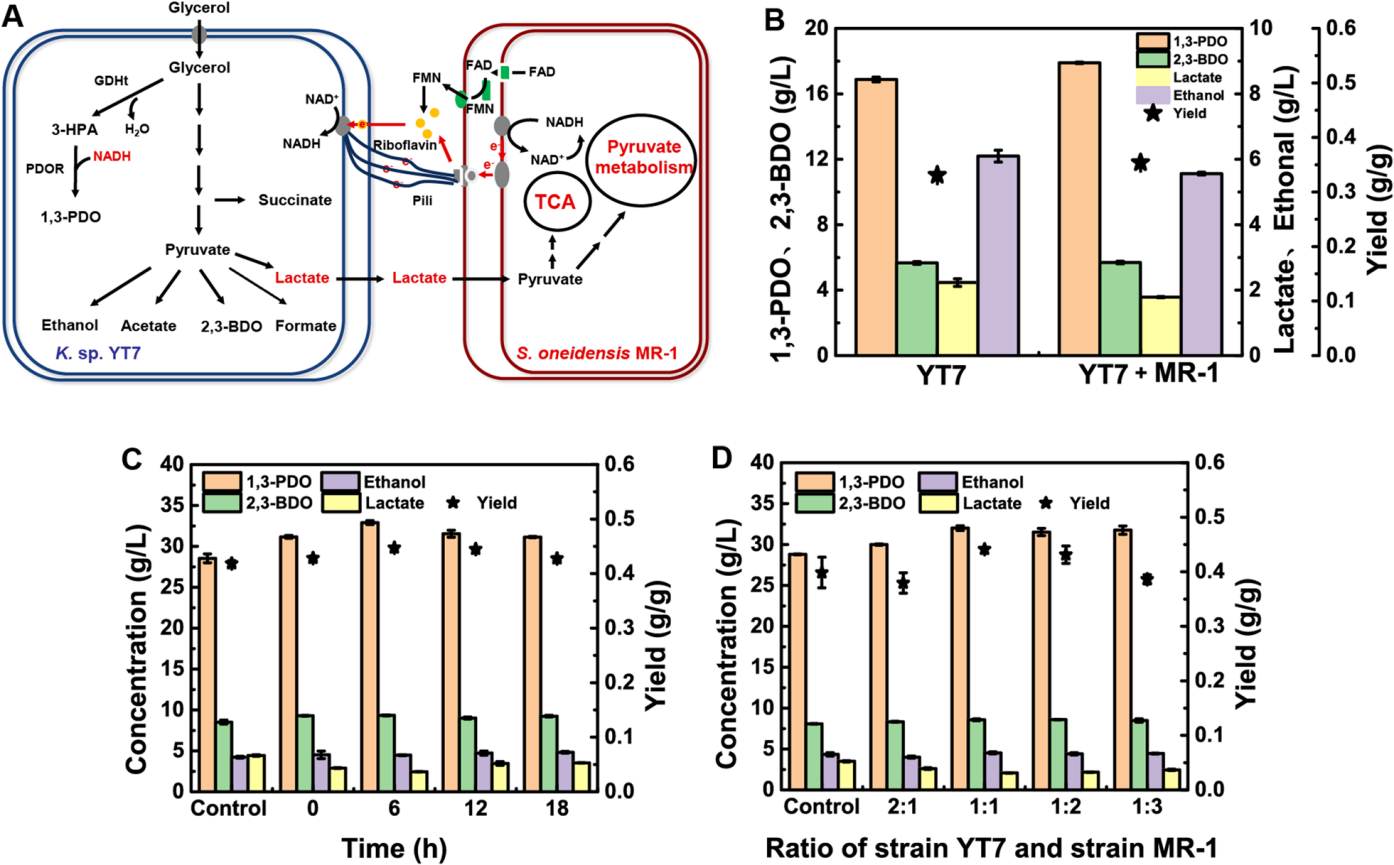
Construction of the microbial co-cultivation system. **A** Substance exchange models in the strain YT7 and MR-1 co-cultivation system. **B** Production of 1,3-PDO and by-products in the co-cultivation system of strain YT7 and MR-1. The inoculation time of strain MR-1 was 6 h after inoculation of strain YT7. The inoculation ratio of strain YT7 and strain MR-1 was 1:1. **C** Effects of different inoculation time on 1,3-PDO production for strain YT7. **D** Effects of different inoculation ratio on 1,3-PDO production for strain YT7

In natural environment, microbial community can improve the intracellular NADH levels through the interspecific interaction to adapt to the environment. To eliminate the supplementation of exogenous electron mediator, an artificial co-culture system was constructed to produce 1.3-PDO by strain YT7 and *S. oneidensis* MR-1. As known, strain MR-1 is an exogenic bacterium that can produce extracellular electron mediator flavins (flavin single nucleotide FMN, riboflavin), which can also metabolize formate, lactate and amino acids. The addition of strain MR-1 in the co-culture system will not only provide electron and electron mediator for strain YT7, but also reduce the concentration of by-product lactate produced in the fermentation process (Fig. 4a).

#### Feasibility test of two-bacterial co-culturing system

In order to verify the feasibility of the co-culturing system, strain MR-1 was cultured in the fermentation medium, and 3 g/L lactate was added to the medium as the carbon source. After 72 h of cultivation, the glycerol in the fermentation medium was not consumed, while 0.76 g/L of lactate was consumed, and OD_600_ of strain MR-1 was increased from the initial 0.512 to 0.752. Although strain MR-1 grew less in fermentation medium, it could consume lactate and did not compete glycerol with strain YT7. Therefore, an artificial two-bacterial co-culturing system can be constructed.

Under the same inoculation time and amount, the titer and yield of 1,3-PDO synthesized by co-culturing system were increased by 6.02% (17.89 g/L) and 6.99% (0.35 g/g) compared with strain YT7, respectively (Fig. 4b). The co-culturing system reduced the accumulation of lactate by 20.02% (1.79 g/L). In addition, the ethanol accumulation was decreased by 8.76% (5.56 g/L). The accumulation of by-product 2,3-BDO did not increase with the increase of 1,3-PDO production (Fig. 4b). The electrons released by electrogenic bacterium MR-1 could trigger metabolic regulation and promote the level of NADH in the mixed bacteria system, while the 1,3-PDO pathway can maintain the intracellular redox balance by consuming NADH [35]. Therefore, the 1,3-PDO process was enhanced by strain YT7 to resist the redox imbalance caused by excessive levels of NADH.

#### Optimization of inoculation time and inoculation ratio

Inoculation time and ratio are two main factors affecting the chemicals production efficiency by microbial consortia. As seen in Fig. 4c, when the inoculation time of strain MR-1 was 6 h after the inoculation of strain YT7, the by-product of lactate in the fermentation medium was the lowest (2.44 g/L), and the 1,3-PDO production was 32.91 g/L (Fig. 4c). The 1,3-PDO titers in the fermentation medium by this microbial consortium were all increased compared to controls without co-cultivation of strain MR-1 (Fig. 4d). When the inoculation ratio was 1:1, the highest 1,3-PDO yield in the fermentation system was 0.44 g/g, and the 1,3-PDO titer was 32.01 g/L. Compared with the control, the titer and yield were increased by 7.63% and 10.85%, respectively (Fig. 4d). The growth of strains YT7 and MR-1 in the co-culture system was detected by dilution coating plate method. The growth of strains YT7 and MR-1 reached 2.41 × 10^8^ CFU/mL and 0.75 × 10^8^ CFU/mL, respectively. In conclusion, it is more favorable to synthesize 1,3-PDO when strain MR-1 was inoculated 6 h later than strain YT7 with the inoculum size of 1:1 (Table 1).

**Table 1:** Comparison of the final product concentration of fermentation in flask

	1,3-PDO (g/L)	2,3-BDO (g/L)	Ethanol (g/L)	Lactate (g/L)
Initial fermentation	11.30 ± 0.98	3.92 ± 0.34	5.41 ± 0.56	3.32 ± 0.31
Fermentation condition optimization strategy	14.63 ± 1.32	5.70 ± 0.51	5.85 ± 0.12	3.04 ± 0.15
Riboflavin addition strategy	28.01 ± 0.16	9.36 ± 0.20	4.04 ± 0.91	3.40 ± 0.83
Two-bacterial co-culturing strategy	32.30 ± 0.28	8.58 ± 0.11	4.53 ± 0.12	2.08 ± 0.08

### Fed-batch fermentation

**Fig. 5:**
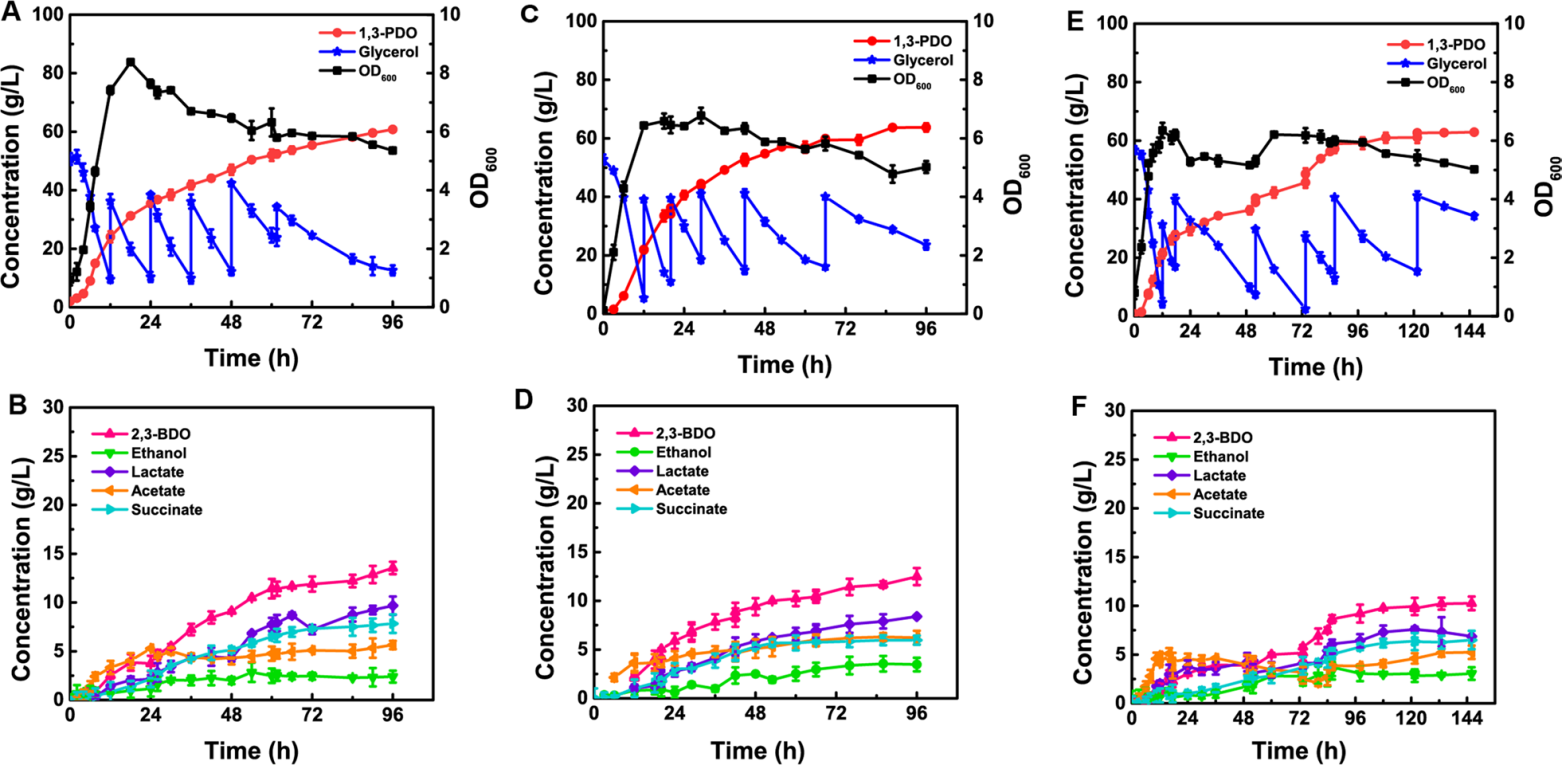
Fed-batch fermentation. Growth and fermentation profiles of *Klebsiella* sp. YT7 in a 5.0-L bioreactor containing mineral salts medium amended with 50 g/L of initial glycerol at 30 °C. **A** Strain YT7 utilizes glycerol to grow and synthesize 1,3-PDO. **B** Strain YT7 utilizes glycerol to synthesize by-products. **C** Strain YT7 utilizes glycerol to grow and synthesize 1,3-PDO with 1 mg/L riboflavin. **D** Strain YT7 utilizes glycerol to synthesize by-products with 1 mg/L riboflavin. Fermentation profiles using a co-cultivation system consisting of *Klebsiella* sp. YT7 and *Shewanella oneidensis* MR-1. **E** Co-culturing system utilizes glycerol to grow and synthesize 1,3-PDO. **F** Co-culturing system utilizes glycerol to synthesize by-products

To further increase the 1,3-PDO production, the fed-batch fermentation was carried out in a 5-L fermentor. When the remaining concentration of glycerol in the fermentor was less than 10 g/L, glycerol was immediately added to 30 g/L. After strain MR-1 was inoculated in the co-culture system, the growth rate of biomass in the fermentation system during 6–12 h was lower than that of strain YT7 alone (Fig. 5a, c, e). During the period of 12–18 h, the biomass showed a downward trend. The biomass of the co-culture system was increased within 42–60 h and then decreased gradually (Fig. 5e). The co-culture system affected the growth of strain YT7 after the inoculation of electricity producing strain MR-1, resulting in the reduction of the synthesis efficiency of 1,3-PDO from glycerol and the prolongation of the fermentation period. The 1,3-PDO titer tended to be stable after 84 h fermentation, and the 1,3-PDO concentration reached 56.23 g/L with the yield and productivity of 0.40 g/g and 0.67 g/(L h), respectively. After 146 h, the 1,3-PDO titer reached 62.90 g/L, which was 3.5% higher than single strain fermentation after 96 h (60.76 g/L), which is also similar to single strain fermentation with 1 mg/L riboflavin (63.73 g/L) (Fig. 5a, c, e). The concentration of by-products generated by co-culture system are generally lower than the fermentation by single bacteria or single bacteria added nuclear lutein (Fig. 5b, d, e). The by-products of 2,3-BDO, ethanol, lactate, acetate and succinate reached 10.26 g/L, 3.06 g/L, 6.86 g/L, 5.23 g/L and 6.49 g/L, respectively. Compared with that by using strain YT7, the concentrations of 2,3-BDO, lactate, acetate and succinate were decreased by 22.82% (13.54 g/L), 29.13% (9.68 g/L), 7.10% (5.63 g/L), 17.01% (7.82 g/L), respectively, however, the acetate concentration was increased by 28.03% (2.39 g/L) (Fig. 5b, e).

## Discussion

1,3-PDO is a versatile platform chemical, which can be used to synthesize polyester, polyurethane and heterocyclic compounds. Compared with chemical methods, the biological synthesis of 1,3-PDO has shown promising applications owning to its mild conditions and environmental friendliness. However, the biological synthesis of 1,3-PDO still has the problem of low titer and yield due to the shortage of reducing powers. In this study, *Klebsiella* sp. strain YT7 was successfully isolated, which can synthesize 11.30 g/L of 1,3-PDO from glycerol in shaking flasks. Actually, *Klebsiella* is the most well studied bacterium for 1,3-PDO production [36]. Many wild-type *Klebsiella* strains have been screened to produce high amount of 1,3-PDO [37, 38]. Moreover, several strategies including metabolic engineering and fermentation engineering have been used to improve the 1,3-PDO titer and yield [36–38].

Currently, the reported optimum growth temperature of *Klebsiella* was 30–37 °C. And most fermentation temperature for 1,3-PDO production by *Klebsiella* was set at 37 °C [26–28]. In this study, strain YT7 showed the highest 1,3-PDO production and relatively low by-products production at 30 °C. As known, glycerol dehydratase and 1,3-PDO oxidoreductase were key catalytic enzymes in the biosynthesis of 1,3-PDO. It is reported that the optimal temperatures for the enzymatic reaction of glycerol dehydration and 1,3-PDO oxidoreductase were 28–45 °C and 30–45 °C, respectively [28–30]. Therefore, it is speculated that *Klebsiella* has high capability to synthesize 1,3-PDO at 28–37 °C, and strain YT7 prefers 30 °C to synthesize 1,3-PDO.

The oxidation–reduction cofactor plays an important role in the metabolic activity of microorganisms, as it is not only an electronic receptor catalyzing the metabolic metabolism in cells, but also provides the restoration for certain oxidation reactions [31]. In terms of the reducing metabolic pathway, 1,3-PDO oxidoreductase can generate one molecular 1,3-PDO with the consumption of two molecules NADH [20, 21]. However, few studies have addressed on the improvement of 1,3-PDO production by the microbial consortia instead of the supplementation of exogenous electron carriers [25]. Therefore, the oxidation–reduction cofactor of NADH/NAD^+^ was further regulated by electron mediators to explore the effects of 1,3-PDO synthesis. The electron mediator can realize electron transmission by carrying electrons to cross the cell membrane, which will affect NADH levels in microorganisms [32–34]. Accordingly, non-physiological (neutral red and methylviologen) electron mediator and physiological electron (riboflavin) mediator were used to improve the level of intracellular reduction force of the strain YT7. Although non-physiological electronic media has a certain inhibitory effect on the growth of the strains, the yield of the strain YT7 is significantly increased by the participation of the electronic media.

Microbial community can improve the intracellular NADH level through the interspecific interaction to adapt to the environment in natural environment. To eliminate the supplementation of exogenous electron mediator, an artificial co-culture system was constructed to produce 1.3-PDO by strain YT7 and *S. oneidensis* MR-1. As known, strain MR-1 is an exogenic bacterium that can produce extracellular electron mediator flavins (flavin single nucleotide FMN, riboflavin), which can also metabolize formate, lactate and amino acids [39]. The electrogenic strain MR-1 and strain YT7 were innovatively constructed as a co-culturing system to improve the 1,3-PDO with glycerol as carbon source (Fig. 4a). Strain MR-1 not only provides electrons for the co-cultivation system, but also metabolizes parts of the by-product of lactate without competing for the substrate glycerol. From the fermentation results, it can be seen that although the production of 1,3-PDO by the co-culture system has been increased, the growth curve of co-culture strains showed that the current co-culture system was still unstable, and needs to be improved through strategies such as material intervention. In nature, microorganisms often exist in the form of mixed bacteria. Inspired by this, the construction of artificial multi-cell culture systems has emerged in the field of synthetic biology in recent years [40]. The method of artificially constructing microbial co-culture system can reduce the concentration of by-products and improve the synthetic efficiency of products. For instance, Daria et al. co-cultured *Alcaligenes faecalis* JP1 and *Clostridium butyricum* DSP1 for 1,3-PDO synthesis [41]. In this system, the 1,3-PDO yield and productivity were increased to 0.53 g/g and 1.07 g/(L h), respectively, and the only by-product butyrate was less than 1 g/L [41]. However, the selection of appropriate host allocation metabolic pathway still faces many engineering problems, such as the complex subspecies dynamics/interaction, short stable production time, and unsatisfactory culture of microbial community and so on [42]. Future prospects are to further optimize the artificial co-culture strategy, improve the stability of the co-culture system, and give full play to the advantages of the co-culture system in increasing production and reducing by-products.

## Conclusion

Biological synthesis of 1,3-PDO has become a hot spot in research and development due to its mild conditions and environmental friendliness. However, the biological production of 1,3-PDO has the problems of insufficient supply of reducing power, lower titer and yield. In this study, *Klebsiella* sp. strain YT7 was isolated, which can synthesize 11.30 g/L of 1,3-PDO with glycerol as the substrate. An intracellular redox level regulation strategy based on the addition of electron mediators was used for fermentation regulation, which increased the 1,3-PDO yield by 147.88%. A two-bacterium co-culture system with *Klebsiella* sp. strain YT7 and *S. oneidensis* strain MR-1 was established, which realized the substitution of exogenous electron mediators and the reduction of by-product accumulation. The titer of 1,3-PDO reached 32.30 g/L with an increase of 185.84%, while the lactate content in the co-culture system was reduced by 38.82%. The synthesis of 1,3-PDO mediated by co-culture system provides a feasible strategy to improve the titer and reduce the concentration of by-products.

## Methods

### Strains and media

*Klebsiella* sp. YT7, isolated from the riverside silt in Nanjing Laoshan National Forest Park, was grown on a seed medium contained 20 g/L of glycerol, 4.45 g/L K_2_HPO_4_·3H_2_O, 1.3 g/L KH_2_PO_4_, 2 g/L (NH_4_)_2_SO_4_, 0.4 g/L MgSO_4_·7H_2_O, 1 g/L yeast extract, 2 g/L CaCO_3_,1 mL of trace element solution. The fermentation medium contained 4.45 g/L K_2_HPO_4_·3H_2_O, 1.3 g/L KH_2_PO_4_, 2 g/L (NH_4_)_2_SO_4_, 1.6 g/L MgSO_4_·7H_2_O, 4 g/L CaCO_3_, 1 mL/L trace element solution. The trace element solution was composed of 5 g/L FeSO_4_·7H_2_O, 2 g/L CaCl_2_, 0.14 g/L ZnCl_2_, 0.2 g/L MnCl_2_·4H_2_O, 0.12 g/L H_3_BO_3_, 0.4 g/L CoCl_2_·6H_2_O, 0.04 g/L CuCl_2_·2H_2_O, 0.05 g/L NiCl_2_·6H_2_O, 0.07 g/L Na_2_MoO_4_·2H_2_O. *Shewanella oneidensis* MR-1 (ATCC 700550) was grown on Luria–Bertani (LB) medium. All media were sterilized at 121 °C for 15 min. Neutral red, methylviologen and riboflavin were sterilized by filtered with a 0.22 μm Millipore membrane.

### Culture conditions

To screen bacteria capable of producing 1,3-PDO, ten soil samples were collected from silt in different areas of Nanjing Laoshan National Forest Park, China. The samples were added to anaerobic bottles containing 50 mL of seed medium containing 20 g/L glycerol was used as carbon source. The culture was incubated at 37 °C on a rotary shaker at 180 rpm for 48 h. Five milliliters of the enrichment culture were then transferred to fresh seed medium every 48 h for subculture. After 3 rounds of enrichment, the different colonies were picked into fermentation medium using glycerol as the carbon source for detection of 1,3-PDO production by spreading serially diluted enrichment cultures. Eventually, the strain YT7 with the best ability to produce 1,3-PDO was obtained. Strain YT7 was aerobically cultured at a rotation speed of 180 rpm and a temperature of 37 °C for 7–8 h. Then, the seed liquid was inoculated to the fermentation medium at a rate of 10% (V/V) for anaerobic fermentation at a speed of 180 rpm and a temperature of 30 °C for 72 h.

Fed-batch fermentation in a 5-L stirred reactor (T&J Bio-engineering (Shanghai) Co., LTD, Shanghai, China) was carried out at 30 °C for 96 h with the inoculation of 10% (v/v) under anaerobic condition, the agitation speed of 250 rpm. The initial glycerol concentration was 50 g/L and was maintained between 10 and 30 g/L by feeding glycerol into the reactor throughout the fermentation process. The pH value was maintained at 7.0 by automatic addition of 4 M NaOH.

### Analytical methods

Cell growth was determined by measuring the optical density at 600 nm. The glycerol and products of 1,3-PDO, 2,3-BDO, lactate, acetate, succinate and ethanol in the fermentation broth were analyzed by high performance liquid phase chromatography (HPLC) (Thermo Fisher Ultimate 3000) equipped with a Shodex RI-101 Detector and an Aminex^R^ HPX-87H ion exclusion column (Bio-Rad) at a flow rate of 0.6 mL/min and a column temperature of 55 °C. The mobile phase was 5 mM H_2_SO_4_. Samples were filtered through 0.22 μm Millipore membrane before analysis.

## Data Availability

All data generated or analyzed during this study are included in this published article.
